# Health of Countries

**Published:** 1874-10

**Authors:** 


### HEALTH OF COUNTRIES.

Every intelligent person ought to have some idea of the relative health of different countries, and different sections of his own country ; this can be the more speedily done by having fixed in the mind the standard or want of health, above or below which makes a locality more or less sickly than the average, as above or below zero enables one speedily to understand the state of the weather. It is a very rare thing that among a thousand persons in any locality, and of all ages and conditions, less than twelve die every year, the number dying in New York City in the last week of July, 1874, was at the rate of forty-four out of a thousand ; but, taking the year round, the average death rate of New York City may be set down at about thirty, showing that nearly three times as many die every year in the metropolis of America as ought to do—that is, three persons die where one ought to ; that three graves are dug where only one should be ; that two persons out of three die prematurely, die before their time ; would not die if proper care were taken ; and proper care means to live cleanlily, eat regularly, work with a steady industry, and get all the sleep the system will take. The English live longer than the Scotch, the Irish live longer than the English. The death rate in Scotland is twenty-four in a thousand, in England twenty-three, in Ireland twenty, in London twenty-two, showing that the greater intelligence and the less exposure to the inclemencies of out-doors, make the city healthier than the country. The death rate of Great Britain is about the same as that of the United States.

